# The Sociocognitive Origins of Personal Mastery

**DOI:** 10.1177/00221465231167558

**Published:** 2023-05-02

**Authors:** Gordon Brett, Soli Dubash

**Affiliations:** 1 University of Toronto, Toronto, ON, Canada; 2 University of Hong Kong, Hong Kong SAR

**Keywords:** cognitive styles, dual-process models, health, mastery, stress

## Abstract

This article examines the relationship between cognitive processing and mastery. While scholars have called for the integration of sociological and cognitive analyses of mastery, sociological research has focused almost exclusively on mapping its social correlates. As a result, sociologists have relied on untested and underspecified assumptions about cognition to explain the efficacy of mastery. Taking an interdisciplinary approach integrating research on mastery, dual-process models of cognition, and intersectionality, we specify and test the hypothesis that deliberate thinking dispositions are associated with a greater sense of control over one’s life chances and assess whether this relationship varies across different intersections of social positions. Regression results from survey data in a diverse student sample (N = 982) suggest a positive correlation between deliberate cognitive style and personal mastery. However, results from a quantitative intersectional analysis demonstrate that this relationship does not hold for East Asian women.

Among the most robust findings from research on stress processes is that mastery—the extent to which people regard their life chances as being under their own control instead of being fatalistically ruled (Pearlin and Schooler 1978:5)—holds considerable significance for health and subjective well-being (Pearlin 1999; Ross and Mirowsky 1989; Ross and Sastry 1999; Wheaton 1980, 1983). Four decades of social scientific evidence has established mastery as consequential for mental health (Louie 2019; Ross and Mirowsky 2013; Thoits 2010), physical health (Gecas 1989; Mirowsky and Ross 2003), health behaviors (Mirowsky and Ross 1998; Pearlin et al. 2007; Seeman and Seeman 1983), and mortality (Roepke and Grant 2011; Tung et al. 2021). Primarily, greater personal mastery affects health by encouraging an active, problem-solving orientation toward the world, in contrast with fatalistic individuals, who are more passive and emotion-focused in coping with life challenges (Conger et al. 2009; Pearlin et al. 2007; Pearlin and Schooler 1978; Thoits 1994; Wheaton 1980). Through this active orientation, mastery mediates and moderates the linkages between the circumstances of everyday life and the deleterious consequences of stress and strain (Pearlin and Bierman 2013; Thoits 1995, 2006, 2010; Wheaton 1983). As a result, social differences in mastery are a life-or-death issue.

Mastery’s considerable consequences for health and well-being continues to provoke a wide range of social scientific research aimed at better understanding where it comes from and how it develops. As Haidt and Rodin (1999) argued, the sense of control should be viewed in a broad, interdisciplinary framework bridging biological (motivational), psychological (cognitive), and sociological (structural) perspectives. However, sociologists have almost exclusively analyzed the sociological correlates of mastery (see Conger et al. 2009; Falci 2011; Mirowsky and Ross 1983, 1992; Pearlin et al. 2007; Ross and Sastry 1999; Schieman and Narisada 2014) separate from the cognitive traits and dispositions likely to inform the development and social stratification of this crucial stress-buffering resource. Instead, sociologists rely on relatively untested assumptions regarding the greater mental effort associated with mastery and use them to explain its effects on behavior and downstream health outcomes (e.g., Mirowsky and Ross 1990, 1998; Thoits 1994, 2006, 2010).^1^ We take seriously Haidt and Rodin’s (1999) claim regarding the synergy derived by taking (in this case) a psychological and sociological approach in tandem and test the presumed relationship between cognitive processing and mastery.

To investigate this relationship, we engage with sociological and psychological research on dual-process models of cognition (Evans and Stanovich 2013; Leschziner 2019; Lizardo et al. 2016). This scholarship separates automatic cognitive processes that are low effort and execute autonomously from deliberate cognitive processes that are high effort and require controlled attention (Evans and Stanovich 2013; Leschziner 2019). Although the capacity for both automatic and deliberate cognitive processes is universal, there is variation in our propensities for these processes—referred to as “cognitive styles” or “thinking dispositions” (Cacioppo, Petty, and Kao 1984; Stanovich 2010)—that are both socially patterned (Brett and Miles 2021) and consequential for health in their own right (Martinelli and Veltri 2021; Tutić, Krumpal, and Haiser 2022). Based on this dual-process approach and extant sociological research on mastery, we operationalize greater mental effort as a deliberate cognitive style and hypothesize that it will be positively associated with mastery.

We test this hypothesis with (to our knowledge) the only publicly available data measuring both mastery and cognitive styles: the Peel Social Lab Survey. Then, drawing on recent advances in the quantitative application of intersectionality theory (Bauer 2014; Bauer and Scheim 2019; Evans 2019), we test for the social stratification of this relationship across six unique positions cross-cutting ethno-racial and gender identities. We find that a deliberate cognitive style is positively associated with mastery but that this relationship is contingent on intersectional social location and does not hold for all intersectional groups. Specifically, we find that when East Asian women report a more deliberate cognitive style, they also report lower personal mastery.^2^ We suggest that deliberate cognitive styles support mastery because greater motivation to engage in effortful cognitive activity facilitates more successful problem-solving, which in turn provides a greater sense of control. However, we suggest that this relationship diverges for East Asian women in our sample as a result of both institutional discrimination (in which their deliberate thinking efforts are disregarded) and issues of institutional fit (where their deliberate thinking is rooted in a chronic state of unsettledness), both of which produce a negative association between deliberate thinking and mastery. These analyses offer a baseline to inform future research testing the causal linkages between mastery and dual-process models of cognition for the stress and health of marginalized intersectional groups.

## Background

### Causes and Consequences of Mastery

Mastery is the generalized belief or understanding individuals hold regarding their ability to manage the circumstances of their lives. Mastery is closely related to constructs such as locus of control (Rotter 1966), self-efficacy (Bandura 1977; Gecas 1989, 2003), planful competence (Clausen 1993), and personal control (Ross and Mirowsky 1989, 2013; Ross and Sastry 1999), all of which reference personal control over life circumstances and are used to measure the subjective dimension of agency (Hitlin and Kwon 2016; Mirowsky and Ross 2007; Pearlin et al. 2007; Skinner 1996). Mastery is critical to health, primarily because of its “stress buffering” function that weakens the impact of stressors (Pearlin 1999; Pearlin and Bierman 2013; Thoits 2010). For those with a greater sense of control, the challenges and stressors in their lives appear less threatening and ominous (Pearlin and Schooler 1978; Roepke and Grant 2011). Rather than passively responding to life challenges, those with a greater sense of mastery are believed to adopt an active, problem-solving approach to life, persevering in the face of challenges or taking the initiative to plan for and avoid them (Caplan and Schooler 2007; Clausen 1993; Conger et al. 2009; Mirowsky and Ross 1990; Pearlin 1991; Thoits 1994, 2006; Wheaton 1980).

Given its far-reaching consequences for stress and well-being, social scientists have worked to establish an array of predictors of mastery related to social location and life course experiences of both control and structural helplessness (Pearlin et al. 2007; Skaff, Pearlin, and Mullan 1996). Both ascribed statuses (e.g., gender, race or ethnicity) and achieved statuses (e.g., social class) contribute to social differences in mastery.^3^ For example, Asians report lower average sense of control than non-Asians (Narisada and Schieman 2016; Sastry and Ross 1998), Black people have a lower sense of control than White people on average (Mirowsky, Ross, and Van Willigen 1996; Ross and Mirowsky 1989), and mastery is lowest for Black people with dark skin tone (Louie 2019). There is, however, highly heterogeneous evidence of the stratification of mastery across genders: Women generally report lower levels of mastery than men, yet recent nationally representative American samples indicate that these differences may be decreasing (Mirowsky 1996; Mirowsky and Ross 1983; Pearlin et al. 2005; Ross and Mirowsky 1989; Thoits 1987). Age is often, but not always, negatively related to sense of control and is likely nonlinear (Robinson and Lachman 2017; Ross and Mirowsky 2002; Schieman 1997).

In terms of achieved statuses, socioeconomic status is positively associated with the sense of control, including family income, education, and employment (Conger et al. 2009; Falci 2011; Mirowsky and Ross 1983, 2003; Moilanen and Shen 2014; Ross, Mirowsky, and Pribesh 2001; Schieman, Nguyen, and Elliott 2003). In general, because mastery beliefs develop through experiences of failure or success in affecting the outcomes of a situation, those in positions of power (e.g., higher earnings, more job autonomy) possess a greater sense of control than those in positions of dependency and with limited resources (Ross and Mirowsky 2013; Thoits 2006; Wheaton 1980). Notably, it is likely that the relationship between mastery and positions of power is bidirectional, with power leading to a greater sense of mastery and mastery leading to achievable positions of power.

While sociologists have thoroughly analyzed the sociological predictors of mastery, they make plausible but relatively untested assumptions regarding the cognitive processes associated with mastery.^4^ Many of these assumptions are embedded in the conceptualization of “active and attentive problem solving” (Ross and Mirowsky 1989:207; see Conger et al. 2009; Thoits 2006) that scholars posit as a general orientation that is both associated with mastery and is used explain the positive effects of mastery on health and well-being. In general, mastery is associated with greater mental and behavioral effort in things such as problem-solving, coping, and goal attainment (see Hitlin and Johnson 2015; Mirowsky and Ross 1998; Wheaton 1980). For example, people with a greater sense of control are said to think more about their problems and figure out their causes, proactively find ways to solve these problems and minimize their negative consequences, and demonstrate forethought in avoiding them in the future (Ross and Mirowsky 1989; Ross and Sastry 1999). Similarly, these active problem-solving efforts are often described as “conscious,” “intentional,” and “deliberate” choices and decisions that constitute exercises in personal agency (Thoits 1994:146, 1995:63, 2006:309–10).

However, neither the construct of mastery nor sociological research on mastery directly assess these reflective and prospective dimensions of agency (see Hitlin and Johnson 2015). As such, although sociologists claim that individuals with a greater sense of control exert more mental effort by “seeking information” to guide their behavior (Mirowsky and Ross 1998:419) and “searching the environment” for potential sources of distress (Ross and Mirowsky 2013:394), they do so with little direct evidence or broader theoretical consideration for the cognitive processes that are implied. To understand how these processes may reflect a disposition for deliberate thinking, we turn to dual-process models of cognition and more specifically to the concept of cognitive styles.

### Dual-Process Models and Cognitive Styles

Dual-process models have been used within cognitive and social psychology for the past several decades and are becoming increasingly influential across the social and behavioral sciences (Evans and Stanovich 2013; Lizardo et al. 2016). These models hold that there are two general types of cognitive processing. Type 1 processes (what we refer to as *automatic* cognition) execute autonomously, make minimal demands on working memory, and are often fast, intuitive, and unconscious. Type 2 processes (what we refer to as *deliberate* cognition) require controlled attention, place greater demands on working memory, and are often slower, deliberative, and conscious (Evans and Stanovich 2013).

While everyone has the capacity to think using automatic and deliberate cognition, there are systematic differences in how often people use these processes. One of the primary reasons for these differences is that some people develop a habit for more automatic or deliberate thinking, which, through frequent repetition over time, becomes their default mode of information processing (see Brett 2022). These differences are what psychologists refer to as thinking dispositions or cognitive styles: general propensities in the reliance on either automatic or deliberate cognition (Cacioppo et al. 1984; Epstein et al. 1996; Frederick 2005). Individuals with an analytical (i.e., deliberate) cognitive style tend to think more broadly and extensively about problem-solving and will examine and critically evaluate their intuitions, while those with an intuitive (i.e., automatic) cognitive style are more likely to trust their initial responses produced by automatic processes (Pennycook et al. 2012; Stanovich 2010).

Differences in cognitive style are tied to social and cultural characteristics suggesting that they are (at least partially) learned. Based on meta-analyses across multiple measures of cognitive style, men are more analytical and less intuitive than women (Brañas-Garza, Kujal, and Lenkei 2019; Brett and Miles 2021), education is positively associated with analytical cognitive style (Brett and Miles 2021), and age is a significant but nonlinear predictor of both intuitive and analytical cognitive style (Brett and Miles 2021; Phillips et al. 2016). Additionally, individuals from collectivist cultures tend to think more holistically, which is characterized by associative thinking, pattern matching, and a preference for intuition. Conversely, people in individualistic cultures typically prefer an analytical cognitive style in which decisions are based on formal, decontextualized rules (Buchtel and Norenzayan 2009).

Although there are a variety of measures of cognitive style (see Epstein et al. 1996; Frederick 2005), there are striking similarities between the cognitive processes and tendencies described by research on mastery and what psychologists refer to as the *need for cognition*: stable individual differences in the motivation to engage in effortful cognitive activity (see Cacioppo et al. 1996; Cacioppo and Petty 1982). The need for cognition is defined by a bipolar continuum of individual differences between chronic cognitive misers (i.e., those with automatic cognitive styles) and chronic cognizers (those with deliberate cognitive styles). People with a low need for cognition tend to solve problems and make sense of the world using heuristics or by relying on others, while those high in need for cognition “tend to seek, acquire, think about, and reflect back on information to make sense of stimuli, relationships, and events in their world” and as a result, possess “richer behavioral histories of cognitively effortful endeavors and effective problem solving” (Cacioppo et al. 1996:198). The active information seeking and effortful problem-solving characteristics of those high in the need for cognition are virtually identical to the cognitive and behavioral tendencies attributed to those high in mastery.

### The Relationship between Mastery and Deliberate Cognitive Style

Both mastery and cognitive styles have been demonstrated to be relatively stable across time and context (see Sadowski and Gulgoz 1992; Stagnaro, Pennycook, and Rand 2018; Wolinsky et al. 2004). However, because they are derived through experience, neither construct is invariant, changing gradually with age. Research suggests that after we have entered adulthood, our propensity for deliberate thinking slowly declines (Brett and Miles 2021; Sladek, Bond, and Phillips 2010), while levels of mastery change relatively little until it declines substantially in old age (Gecas 1989; Mirowsky 1995). Although there is some evidence that negative shocks (e.g., the health and economic shocks caused by COVID-19) can impair cognitive function, thereby reducing deliberate cognitive style (Bogliacino et al. 2021), most changes in both mastery and cognitive style appear to occur slowly and incrementally over time through the gradual accumulation of relevant experiences.

In theory, neither mastery nor deliberation are *necessary* for the development of the other. In our encounters with the world, people work through and overcome situational challenges through either habitual or deliberate modes of thought and action (see Joas 1996). In principle, one can intelligently achieve goals, overcome challenges, and successfully move through life by employing existing habit and skills or relying on intuition to develop a sense of mastery with little recourse to deliberation. Conversely, deliberate thinking can take a variety of forms (recollection, calculation, hypothetical thinking, imagination) and produce a variety of behavioral outcomes (problem-solving, inaction, avoidance, imitation) that may or may not contribute to our overall sense of control.

However, based on research on both mastery and the need for cognition, we suspect that their association is grounded in effective problem-solving. Those with a need to think deliberately about the issues and challenges they encounter are more successful problem solvers on average (see Nair and Ramnarayan 2000), and mastery increases when people successfully solve their problems. This is because beliefs about our sense of control are often realistic, learned through successful or unsuccessful attempts at affecting the outcome of a situation (Ross and Sastry 1999; Wheaton 1980). Therefore, as a result of their successful problem-solving, deliberate thinkers are more likely to see a causal link between their own choices and efforts and their desired outcomes, gradually developing a generalized belief in their ability to control their circumstances and a more proactive approach to life. In general, then, deliberate thinking should be associated with mastery because it provides a more effective way for people to influence their own lives.

*Hypothesis 1*: Deliberate cognitive style is associated with a greater sense of control over one’s life chances.

Inspired by the theoretical tenets of intersectionality (Bowleg 2012; Crenshaw 1991), we also suspect that the relationship between cognitive styles and mastery varies across social positions of power. However, theories on power and information processing offer conflicting predictions regarding this relationship (see Fiske 1993; Keltner, Gruenfeld, and Anderson 2003; Smith and Trope 2006). Approach inhibition theory (Keltner et al. 2003) suggests that power is associated with automatic processing because powerful people have greater resources and opportunities that activate approach tendencies associated with automatic social cognition; they pursue goals with less concern for the social consequences of their actions, are less attentive toward others, and rely heavily on snap social judgments using heuristics such as stereotypes (see Fiske 1993). Conversely, a lack of power is associated with deliberate processing because those with little power have limited resources and face greater amounts of threat and punishment, activating tendencies associated with inhibition and controlled social cognition, specifically, vigilance towards threat, deliberate social judgments, and greater scrutiny of the actions and intentions of others.

By contrast, construal level theory (Trope and Liberman 2010) proposes that power is associated with deliberate thinking. Construal level theory argues that we mentally transcend our immediate experiences and situations by forming abstract mental representations (“construals”) of psychologically distant objects that we use for memory, prediction, and speculation (see Trope and Liberman 2010). As psychological distance (in terms of time, space, social distance, and hypotheticality) increases, people use more abstract representations of objects. This theory suggests that because social power engenders a sense of distinction and therefore psychological distance from other (less powerful) people, it also predisposes them to more abstract thinking and therefore more reflective information processing (see Smith and Trope 2006). Conversely, people with less power rely on more concrete thinking and more specific representations without reflecting on the “bigger picture.”

Although these theories generally conflict, they offer potentially complementary frameworks for understanding how the relationship between deliberate thinking and mastery varies across social positions of power. Approach inhibition theory suggests that the increased deliberation of those with little power is grounded in vigilance and narrowed attention toward threats, including those related to social aggression, racism, and discrimination (Keltner et al. 2003:269), and results in inhibited behavior (hesitation, passivity, and withdrawal). Based on these social circumstances, deliberate thinking promotes anxiety rather than mastery and inactivity rather than proactivity. Therefore, we expect that the deliberation of those from marginalized groups will lead to a decrease in their sense of control. Conversely, construal level theory’s argument regarding the link between positions of power and abstract thinking suggests that this relationship may reinforce the already elevated sense of control of those in power. Because abstract thinking is more flexible and less constraining, it also increases the subjective sense of power and control, encourages forethought, and makes the powerful more likely to act in pursuit of their long-term goals (Smith and Trope 2006; Smith, Wigboldus, and Dijksterhuis 2008). As such, we expect that the deliberate thinking of those in positions of power will lead to even greater increases in the sense of control.

*Hypothesis 2*: The relationship between deliberate cognitive style and mastery will vary across different intersections of social positions, with a positive interaction for people in positions of power (e.g., White men) and a negative interaction for those from marginalized groups (e.g., Asian women).

To test this hypothesis requires an analysis of the social locations that potentially condition the relationship between deliberate cognition and mastery. Informed by the extensive social research on the importance of intercategorical statuses (e.g., gender, race, or ethnicity) for mastery as we reviewed previously, we take an explicitly quantitative intersectional analysis to consider how the focal relationship may be contingent on cross-cutting identity categories (Bauer 2014; Bauer and Scheim 2019; Evans 2019). Recognizing that social categories such as race and gender are overlapping and interactive rather than exclusive categories of experience and analysis (Collins 1991; Crenshaw 1991), we do not “control” for gender or race-ethnicity. Instead, we model the multiplicative potential for our focal relationship to vary by the intercategorical statuses of each gender and race in the sample. Indicated by the intersectional differences demonstrated in Table 2 and the accompanying figures, models that instead “control” for the intercategorical statuses with a simple White or non-White dummy variable would have homogenized the linkages between deliberate cognitive processes and personal mastery and explained less of the total variation in mastery.

## Data and Methods

### Study Sample

The present study tested the hypothesized association between cognitive styles and mastery through a secondary data analysis using the only publicly available data measuring both mastery and cognitive styles: the 2019 to 2020 first wave of the Peel Social Lab Survey (PSL). A unique strength of this study is the large sample from a diverse Canadian university. Respondents were two cohorts of first-year undergraduates who, encouraged to participate with one bonus percentage in a large introductory course, indicated informed consent and then answered a battery of questions. Pooling these cross-sectional cohorts increased statistical power to provide for the testing of both the hypothesized association linking cognitive styles and mastery and an interrogation of potentially unique differences across self-reported gender and ethno-racial identities. To reflect which cohort the respondents participated in, all models included a dummy variable. The order in which respondents answered questions within each section was randomized to mitigate question order bias. The survey responses were anonymized, and respondents who did not consent to their information being used for research purposes were removed from the public release prior to this secondary data analysis. Variables truncated or removed in the process of anonymizing the data for public release held no theoretical relevance for the hypotheses under investigation. After missing data were handled, the analytic sample consisted of 982 respondents.

### Measures

#### Mastery

These data contained three items from the Pearlin and Schooler (1978) mastery scale. Each respondent was asked how strongly they agreed or disagreed with the following statements (“strongly disagree” = 1, “disagree” = 2, “neither agree or disagree” = 3, “agree” = 4, “strongly agree” = 5). The statements were: (1) “I have little control over the things that happen to me,” (2) “I often feel helpless dealing with the problems of life,” and (3) “Sometimes I feel that I’m being pushed around in life.” In seminal research (e.g., Pearlin and Schooler 1978), these three items had the highest factor loadings from the full seven-item scale. Following established practices, we reverse-coded these items and then used the arithmetic mean to create a scale with higher scores indicating greater levels of mastery (α = .71).

#### Cognitive style

Our focal independent variable was three items from the Need for Cognition (NFC) scale. This is a validated self-report measure of preferences for analytical processing that predicts behavior in ways that are consistent with dual-process theories (Epstein et al. 1996). For example, analytical thinkers produce slower response times, rely less on heuristics, and are less susceptible to framing effects than intuitive thinkers (Björklund and Bäckström 2008; Epstein et al. 1996; Gambetti et al. 2021; Pacini and Epstein 1999). This widely used scale measured cognitive styles by asking respondents to indicate how much each of the following statements represents them from “definitely not true of myself” (0) to “definitely true of myself” (4).

Using only a few items to measure deliberate cognitive style is not uncommon. For example, the REIm–13, a validated, shortened version of the Rational-Experiential Inventory, measures rational thinking using a four-item subscale (see McGuiness et al. 2019). Respondents were asked if they “enjoy problems that require hard thinking,” an item that has consistently high loadings in the NFC scale (see Cacioppo and Petty 1982; Epstein et al. 1996). They were also asked if they “enjoy intellectual challenges” and “prefer my life to be filled with puzzles that I must solve,” two of the four questions included in the rationality subscale of the REIm-13. We took the mean of these three items to create a scale with higher scores indicating greater preference for deliberate processing (α = .79).

#### Covariates

Respondents also reported several ascribed and achieved statuses known to be associated with social differences in mastery. Gender was coded as 1 for men and 0 for women. Race-ethnicity was coded with three dummy variables: South Asian (reference), White, and East Asian. Employment status was coded as 0 for respondents not working and 1 for respondents currently working for pay. The education level of caregivers contrasted at least one caregiver in the household having attained a university degree or higher coded as 1 with both caregivers having less than a bachelor’s degree when respondent was 16 years of age (coded as 0). Cohort was coded as 1 for the 2020 cohort.

### Analytic Strategy

**Figure 1.**
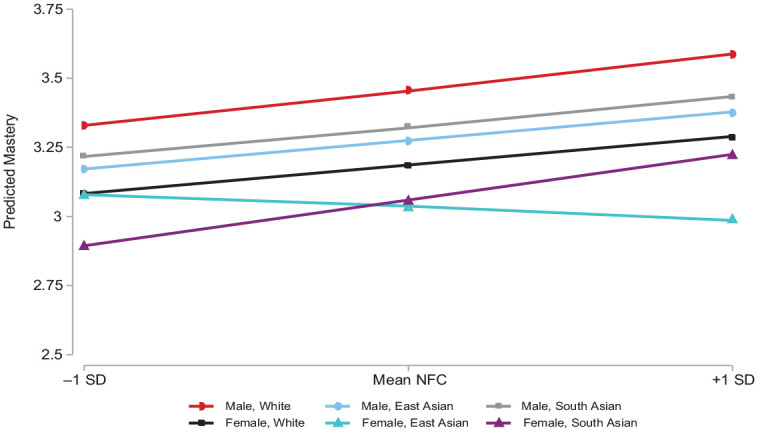
Mastery and Deliberation across Intersectional Groups. *Source*: Peel Social Lab 2019 to 2020 data (*N* = 982). *Note*: All control variables are held constant at mean levels. SD = standard deviation; NFC = need for cognition.

**Figure 2.**
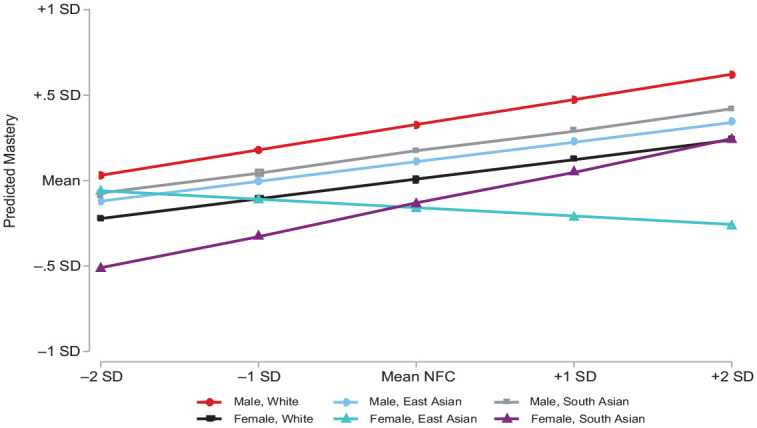
Mastery and Deliberation across Intersectional Groups, Standardized. *Source*: Peel Social Lab 2019 to 2020 data (*N* = 982). *Note*: All control variables are held constant at mean levels. SD = standard deviation; NFC = need for cognition.

The analytic advantage of the quantitative intersectional approach is an explicit focus on the myriad social differences linked with ascribed statuses. Briefly beginning with descriptive statistics in Table 1, we turn to testing for heterogeneity in the focal relationship across intersectional ethno-racial and gender identities in Table 2. With regression coefficients in cross-sectional samples best interpreted as descriptive comparisons, this study built up models that allowed for the comparison of six cross-cutting statuses. Taking quantitative intersectionality seriously and following best statistical practice, we created models that used the largest intercategorical subsample as the reference category for all regression models: South Asian Women. In the first model, we regressed mastery on deliberate cognitive style for all people in the sample. The second model evaluated three-way interactions to test for social differences in the main association across positions in cross-cutting social hierarchies. The final model adjusted for all theoretically relevant covariates in these data. Estimates from this final model are graphically depicted in Figures 1 and 2 to aid comparison across intercategorical statuses.

**Table 1.** Description of Study Variables, Peel Social Lab 2019–2020 Data (*N* = 1,133).

	Mean/Proportion	Standard Deviation	Minimum	Maximum	% Missing
Mastery	3.17	.87	1	5	1
Need for cognition	2.33	.87	0	4	5
South Asian women	.28		0	1	3
South asian men	.12		0	1	3
White women	.23		0	1	3
White men	.09		0	1	3
East Asian women	.17		0	1	3
East Asian men	.11		0	1	3
1+ Caregiver university educated	.51		0	1	10
Employed	.28		0	1	0
Cohort	.37		0	1	0

*Note*: Percent missing is rounded to the nearest integer.

**Table 2.** Mastery Regressed on Cognitive Style and Covariates, Peer Survey Lab 2019–2020 Data (*N* = 982).

	Model 1		Model 2		Model 3	
	*b*	SE	*b*	SE	*b*	SE
NFC	.15***	(.03)	.19**	(.06)	.19**	(.06)
Interactions						
NFC × White Women			–.07	(.09)	–.07	(.09)
NFC × East Asian Women			–.23*	(.10)	–.24*	(.10)
NFC × South Asian Men			–.07	(.12)	–.07	(.12)
NFC × White Men			.10	(.19)	.10	(.19)
NFC × East Asian Men			.22	(.19)	.23	(.19)
Intersectional means						
South Asian men			.43	(.28)	.42	(.29)
White women			.31	(.21)	.29	(.21)
East Asian women			.51*	(.23)	.54*	(.23)
White men			–.24	(.51)	–.22	(.51)
East Asian men			–.54	(.46)	–.57	(.46)
Controls						
Cohort			–.10	(.06)	–.10	(.06)
1+ Caregiver university educated					.06	(.06)
Employed					.08	.06)
Constant	2.84***	(.08)	2.65***	(.14)	2.60***	(.15)
R^2^	.021		.055		.058	

*Note*: Robust standard errors are in parentheses. NFC = Need for cognition.

* *p* < .05, **p < .01, ***p < .001 (two-tailed tests).

The highly diverse student population from which this sample was drawn provided for the potential interrogation of our hypothesized focal relationship across 16 (eight ethno-racial identities and two genders) unique intersectional statuses. However, small cell sizes for several of these groups when considering ethno-racial and gender status simultaneously resulted in low statistical power and connoted the inability to detect relatively small group differences, producing imprecise estimates. As a result, we focused on the largest three ethno-racial groups (which have greater than 100 respondents for each gender in this study): White people, South Asian people, and East Asian people. By doing so, we aimed to provide reliable evidence on the social stratification of the hypothesized relationship while taking into account the challenge of small subgroup sizes for quantitative intersectional analyses. Including the full prism of intercategorical statuses did not substantively alter the results presented, nor were three-way interactions for the excluded statuses significantly different from the main association presented herein.

Regression models provided estimates and hypotheses tests while handling missing data and the pooled nature of these data. The amount of missing data was generally low but varied across measures. The percentage for each covariate is reported in the final column of Table 1. Study conclusions were substantively similar when missing data were adjusted for through full information maximum likelihood estimation (FIML; Allison 2012) or multiple imputation with chained equations (MICE; Enders 2001; see link to OSF replication package for comparison table: https://osf.io/ryhtx/). This similarity and the importance of parsimoniously visualizing the three-way interactions required to test our second hypothesis^5^ guided our decision to use complete case analysis instead of FIML or MICE to handle missing data. Additionally, robust standard errors were used in all models to adjust for heteroskedasticity and the clustering of two cohorts (Freedman 2006; Mansournia et al. 2021). This was particularly salient for our analyses due to the second cohort experiencing the macro-level shock of the COVID–19 pandemic. Due to substantial biases associated with using clustered standard errors with fewer than 10 clusters (with the rule of thumb being ≈50 clusters; see Angrist and Pischke 2009; Cameron and Miller 2015), we instead used robust standard errors to account for the pooled nature of these data. We also adjusted for student cohort with a dummy variable to further address potential considerations about differences across clusters (Cameron and Miller 2015:32).

Throughout, estimates can be straightforwardly interpreted to guide policy development, inform future intervention studies, and conduct meta-analytic review. All multi-item scales used the arithmetic mean instead of sum scores. Due to this, a 1-unit increase in a coefficient indicates going from strongly disagree to disagree, for example, on all items of a scale. This is an interpretative advantage over other approaches, such as sum scale construction. We also provide both direct estimates and standardized visualizations to aid in the interpretation of the substantive importance of the association across groups. Following recent simulation studies, we treated these ordinal variables as indicators of continuous latent constructs (Rhemtulla, Brosseau-Liard, and Savalei 2012; Robitzsch 2020). Taking the sum of these scales did not substantively change the conclusions presented in this study, nor did logit models for ordered categorical data. Data processing and modeling used STATA 16.1. A replication package is hosted on the OSF: https://osf.io/ryhtx/.

## Results

With this student sample drawn from the largest university in the most multicultural Canadian city, White-identifying people are not the largest ethno-racial subgroup. Proportions, shown in Table 1, emphasize the importance of investigating the prism of social differences beyond the use of a White or non-White dummy variable. Overall, more respondents identify as women than men in this sample. The largest intersectional group is comprised of South Asian people, followed by White people and East Asian people. Among these respondents, slightly over half lived at age 16 with at least one caregiver having completed postsecondary education. Between one quarter and one third of students were employed when they were surveyed, and 37% of students were recruited from the 2020 cohort.

Table 2 regresses mastery on cognitive styles and covariates. Demonstrating a significant association for the entire sample, Model 1 provides support for our first hypothesis. People reporting a more deliberate cognitive style also endorse, ceteris paribus, higher levels of personal mastery (*b* = .15, *p* < .001). This direct association accounts for approximately 2% (R^2^ = .021) of the overall variation in mastery for these people. Models (in replication package) that exclude cognitive style and only include controls explain less than 1% (R^2^ = .008), and models that adjust for cognitive style and the control covariates without intersectional means or slopes increase that to about 3% (R^2^ = .029). These two alternate model specifications suggest that the hypothesized focal association would make less of a contribution for explaining inequalities in personal mastery without the explicit investigation of quantitative intersectional differences.

Consistent with stress and health literature, social structural location predicts differences in mastery. Overall, South Asian women (the reference group) who report a more deliberate cognitive style also report higher levels of personal mastery (*b* = .19, *p* < .01). People who identify as White or South Asian men do not report a detectibly different mean mastery, or association, from those in this reference group. This suggests that reporting a higher need for cognition also corresponds with a higher belief in one’s personal mastery for most people in this sample. Adjusting for theoretically informed controls in Model 3 does not change this pattern. Figure 1 visualizes the unequal relationship between deliberation and personal control across these statuses. Substantively, Figure 2 indicates that compared to those with a less deliberate cognitive style, those with a higher need for cognition report about one half a standard deviation greater personal mastery. Thus, we find some support for Hypothesis 1.

Interactions and mean differences for unique intersectional statuses are not detectibly different from the focal association for five of the six intercategorical locations.^6^ By contrast, there is a notable exception to the generally positive relationship between deliberation and mastery. Compared with South Asian and White people, East Asian women report higher mean mastery (*b* = .54, *p* < .05) when reporting the same need for cognition. Along with this significant mean difference, East Asian women with a higher need for cognition also report lower levels of personal mastery (*b* = −.24, *p* < .05). Figure 2 shows that highly deliberate East Asian women are expected to report about one quarter of a standard deviation lower personal mastery than those with a low need for cognition. This provides some support for our second hypothesis but does not conform to our overall prediction, raising further questions as to the mechanisms behind this relationship.

Interestingly, and consistent with intersectionality theory, the challenges of veridically investigating cross-cutting social identities are worthwhile inasmuch as they provide additional purchase on understanding the prism of social differences. Doing so, as shown in Table 1, approximately doubles the total variation explained in mastery and, as shown in the accompanying figures, suggests an otherwise hidden pattern that diverges from the sample majority. Having a larger proportion of the variance explained would be desirable for our focal relationship between deliberate cognitive style and mastery. However, the adjusted R^2^ in our final model is comparable with other studies that predict mastery when models adjust for gender and ethno-racial statuses (see Pearlin et al 2007; Schieman and Narisada 2014). Although the R^2^ in our final model is modest, we suggest that the present study still usefully contributes to understanding the social patterning of mastery across intercategorical statuses given our a priori hypothesizing from the literature and the limited range of variables available in these data. Many of these statuses, however, are not detectably different from the focal association.

Decomposing the association across intercategorical identities is an important step when taking the investigation of social differences in resources seriously, yet it is evident that quantitative intersectional scholarship requires relatively large samples due to the complexity of estimating many three-way interactions between the focal predictor, ethno-racial statuses, and genders. This may also be behind the lack of significant gender differences predicted by seminal studies of mastery. However, and as suggested by recent literature, it is also possible that these genders are growing less unequal in regard to personal mastery. Finally, the importance of caregiver education at home when respondents were age 16 and respondent employment status are not significant in these models.

## Discussion

The relationship between mastery, stress, and health is well established within the social sciences. While there are decades of social science research establishing the sociological correlates of mastery, this scholarship has relied on relatively untested assumptions regarding the cognitive processes underpinning mastery as an explanatory mechanism for its causal efficacy. To remedy this, we follow Haidt and Rodin (1999) in combining social and cognitive perspectives on mastery. Drawing on insights from dual-process models of cognition and intersectionality theory, this study predicted and empirically tested a focal relationship between deliberate cognitive styles and mastery and the contingency of this association across ethno-racial and gender identities.

We find a mostly positive relationship between deliberate cognitive style and mastery. There is some evidence, however, that this relationship is contingent on intersectional social identity. Regression estimates indicate that people reporting a more deliberate cognitive style on average also report greater belief in their personal mastery. By contrast, when reporting greater need for cognition, East Asian women also report lower personal mastery. Quantitively applying intersectionality theory approximately doubles the, albeit modest, coefficient of determination to increase the explained variation in mastery. Taken together, these findings both suggest that mastery is related to cognitive processing and that there are social patterns in the cognitive origins of mastery.

Drawing on research on both mastery and cognitive styles, we argue that the positive association between deliberate cognitive style and mastery is grounded in successful problem-solving because people with deliberate cognitive styles should have richer histories of successful problem-solving, which in turn should increase their sense of control (see Cacioppo et al. 1996; Ross and Mirowsky 2013). However, the findings from our quantitative intersectional analysis did not fit neatly with our predictions and require additional explanation.

Bridging intersectionality (Collins 1991; Crenshaw 1991) with psychological research on power and information processing (Keltner et al. 2003; Smith and Trope 2006), we expected that the relationship between deliberate thinking and mastery would vary across social positions of power. However, we found that the relationship only diverged for East Asian women. Given that there was a negative interaction for East Asian women but a positive interaction for South Asian women, both of which constitute multiply marginalized social groups, this suggests that general power disparities within society cannot explain these findings alone. Alternatively, one might look to cultural differences for the explanation; for example, East Asians generally think more holistically compared to their more analytical Western counterparts (see Buchtel and Norenzayan 2009), suggesting that it is because they are engaging in deliberate thinking, rather than their preferred or more conventionally used mode of thought, that their sense of control decreases. However, given that the relationship between deliberation and mastery diverges between East Asian women and East Asian men, our findings likely do not reflect purely cultural variations in the cognitive origins of mastery.

Instead, we suggest that our findings are best explained by applying both cultural and intersectional analysis more narrowly to the institutional context in which these data were gathered, focusing specifically on why increased deliberate thinking decreases the sense of control of East Asian women in the University of Toronto. Although intersectionality is often seen as shaping entire social systems, it can also be used to understand how multiple axes of oppression operate within specific institutional contexts (although approaches to intersectionality vary in the degree to which they grant institutional primacy in explaining the production of social inequalities; see Choo and Ferree 2010). Similarly, while cultural psychologists often focus on cross-national comparison, cultural sociologists examine how the habits, skills, strategies and worldviews acquired and used by actors fit within institutional arrangements (Lizardo 2017; Lizardo and Strand 2010; Swidler 1986). We apply these approaches to contemporary research on East Asian students in North American universities, suggesting that the reflective thinking of East Asian women decreases their sense of control as the result of institutional discrimination and issues of institutional fit.

East Asian students are often lauded for their academic performance; however, they face a variety of barriers to success in North America universities. For our purposes, the most significant institutional barrier is the bias toward talkative students and “active participation” in Western education (see Ma 2020; Xiao 2021). This disadvantages both domestic and international Chinese students, who often possess learning styles based on quiet engagement, intense listening, and “deep thinking” (Li 2012; Ma 2020; Xiao 2021). Relatedly, there is a cultural norm of thinking carefully before speaking, which restricts Chinese students’ ability to speak up and makes them slower and less spontaneous class participants in spite of their motivation to meet expectations around active engagement (Ma 2020). Chinese students also are commonly more silent in class to show respect to instructors and out of a concern over wasting other students’ time, which is often misinterpreted as a lack of engagement (Liu 2001). The result is that Chinese students are penalized for their passive (although more reflective and contemplative) orientation and reduced engagement in an institutional context that favors active participation (Xiao 2021).

We suspect that this has specific and disproportionate impacts on East Asian women. For one, their sense of self and identity is often affected by cultural notions that passivity and inability constitute feminine virtues, which may inhibit them inside and outside the classroom (Xiao 2021:12). Particularly relevant is the virtue of “feminine speech” in East Asian Confucianism, which states that women must “choose words [carefully] when speaking. . . . Speak only when the time is right; then, others will not dislike one’s utterances” (Pang-White 2018:54). Therefore, cultural norms and expectations are much more constricting of East Asian women’s speech than their male counterparts. Second, some ethnographic research finds that East Asian women face distinct forms oppression in Canadian universities: being excluded by classmates, ignored by university faculty, and therefore unable to receive important resources and supports (Liu 2017). This may be particularly frustrating given that at least in the context of U.S. universities, East Asian women were more likely than East Asian men to report putting more effort into studying than American students, and they firmly believe that learning is driven first and foremost by effort (Ma 2020:91). As intersectionality would suggest, experiences of oppression cannot be separated into those due to race and ethnicity and those due to gender but instead must be viewed as “simultaneous and linked” (Choo and Ferree 2010:132).

In realizing that their existing skills, dispositions, and strategies are ill-suited to context of North American universities, we expect that East Asian women engage in a reflective and cognitively costly search for new cultural models with which they may reorganize their behavior (Lizardo and Strand 2010). East Asian students are aware of the Western emphasis placed on more active or verbal classroom participation and recognize the “mismatch” between their habitual strategies of action and the institutional expectations placed on them (Ma 2020). Because of this, the lives of many East Asian women are chronically “unsettled,” and we should expect them to seek out tutors, mentors, and practical texts in search of explicit guidance until they are able to successfully adjust their practical behavior to the expectations of university life (Lizardo and Strand 2010; Swidler 1986).

This suggests that there are two interrelated pathways through which increased deliberate thinking leads to decreased mastery for East Asian women. First, as the result of East Asian women’s particularly careful and contemplative approach to class participation and the disregard they face from fellow students and professors, the lack of reward or recognition they receive for their reflective efforts in class engenders a greater sense of futility with regard to their sense of self-efficacy. Second, the recognition that their passive orientation is misaligned with the reward system in North American universities produces a state of mild “crisis,” which initiates an active and anxious search for explicit models from which to develop new strategies of action. The more prolonged this reflective search, the more intense their experiences of negative affect (Shaw 2021) and their sense of hopelessness.

While we focus primarily on mastery, the findings from this study also have implications for important debates about culture and cognition. Several sociologists have argued that our beliefs and behaviors are grounded primarily in unconscious dispositions that operate through automatic cognition, discounting the role of reflective thought (Miles 2015; Vaisey 2009). Conversely, we find that the propensity toward deliberate thinking predicts the beliefs that people hold about their own agency. This indicates that deliberate cognition indeed plays an important role in how at least some kinds of beliefs work and should caution sociologists from downplaying the significance of reflective processes in cultural analysis. Second, given that cognitive styles predict not only mastery but also moral values and religious, romantic, paranormal, and conspiracy beliefs (see Pennycook et al. 2012, 2014; Swami et al. 2014; Trémolière and Djeriouat 2019), this suggests that they may be an important cognitive mechanism for cultural acquisition (Cerulo, Leschziner, and Shepherd 2021; Lizardo 2017).

Although to our knowledge this study uses the best publicly available data to test the proposed lines of inquiry, there are several limitations to briefly note. Because these analyses are based on cross-sectional data, we cannot make definitive causal claims. Future prospective research with multiple observations per person would increase confidence in the causal nature of the focal relationship in this study (Vaisey and Miles 2017). Furthermore, following best practice, these models only adjust for ascribed statuses that are not theoretically suggested to be mediators or colliders for the focal relationship (Grätz 2022; Westfall and Yarkoni 2016). The inclusion of additional control covariates that were not included in these data, such as age, is a key area for future inquiry. Another limitation to note is that gender-nonconforming people were not polled in these data. Their experiences likely lead to beliefs in mastery and powerlessness that are significantly different than those of men and women. Moreover, our analysis focuses on intercategorical differences in our focal relationship and does not move beyond categories to evaluate relevant variables as continua (e.g., skin tone) along which forms of discrimination differentially operate (e.g., colorism; Louie 2019; Monk 2015). Mirowsky and Ross (1991), additionally show that there are measurement biases (e.g., agreement or defense tendencies) inherent to scales that contain only items with a similar statement valence, such as found in these data. Lastly, because this sample is made up of postsecondary students from a large introductory undergraduate sociology course, it is not statistically representative of the undergraduate student body at this diverse university or the overall population. A key area for future research is to replicate these patterns in a nationally representative sample with a balanced measure of personal control.

## Conclusion

To conclude, although sociological research depicts increased cognitive effort from those with higher levels of mastery, this association has generally been assumed rather than specified and tested directly. Bridging research on mastery with insights from dual-process models of cognition, we expected that deliberate cognitive styles were significantly associated with mastery. Drawing on theoretical tenets and methodological developments from intersectionality, we then hypothesized that this focal relationship would be conditioned on social positions of power. Comparative evidence from cross-cutting ascribed statuses indicates that for most, but not all, of the distinct ethno-racial and gender intersections in this sample, people reporting a more deliberate cognitive style also, ceteris paribus, endorse stronger beliefs in their personal mastery. This study suggest that cognitive styles hold important implications for researchers interested in mastery and its effects on stress and health inequality.

## Notes

1. This is not to say that all sociologists ignore the psychological components of mastery or related constructs (e.g., Clausen 1993; Johnson and Hitlin 2017).

2. When writing about racial and ethnic groups, there are well-reasoned arguments for using both the upper and lower case, related primarily to the neutralization and naturalization of race and racism. In this article we capitalize all ethno-racial labels consistently to indicate the artificiality of racial categories.

3. Notably, some cross-cultural research in psychology suggests that mastery beliefs may operate differently for Asian respondents. According to this research, Westerners have a more independent view of the self and are therefore more likely to understand both themselves and the behavior of others in terms of psychological traits and attitudes, while those from collectivist cultures have more interdependent self-concepts that emphasize roles, relationships, and the situated nature of personality and behavior (for a review, see Henrich, Heine, and Norenzayan 2010). These differences may help explain why Asians have lower perceived control that non-Asians (see Narisada and Schieman 2016; Sastry and Ross 1998) but also suggest that such personal measures of the sense of control may not adequately reflect the embedded nature of the Asian self-concept.

4. This is certainly not unique to sociological research on mastery. At base, much of the research on mastery analyzes the acquisition and use of beliefs, processes that are necessarily cognitive and that most sociologists cannot discuss without making some minimal assumptions about how cognition works (see DiMaggio 1997; Lizardo 2017; Lizardo et al. 2020).

5. We thank an anonymous reviewer for this suggestion.

6. There is some evidence that these groups may have substantive differences when compared to South Asian women both through the visualization of the mean and slope differences in Figure 1 and as substantiated by extant literature.
